# Quantifying the influences of radiation therapy on deformability of human red blood cells by dual-beam optical tweezers†

**DOI:** 10.1039/d1ra01948a

**Published:** 2021-04-27

**Authors:** Medine Tuna Inanc, Irem Demirkan, Cemile Ceylan, Alper Ozkan, Ozcan Gundogdu, Utku Goreke, Umut A. Gurkan, Mehmet Burcin Unlu

**Affiliations:** Department of Physics, Bogazici University Bebek Istanbul 34342 Turkey burcin.unlu@boun.edu.tr; Istanbul Oncology Hospital Maltepe Istanbul 34854 Turkey; Health Sciences Institute, Yeditepe University Istanbul Turkey; Biomedical Engineering, Kocaeli University Kocaeli Turkey; Department of Mechanical and Aerospace Engineering, Case Western Reserve University Cleveland Ohio USA; Department of Biomedical Engineering, Case Western Reserve University Cleveland Ohio USA

## Abstract

Radiation therapy is widely used as a treatment tool for malignancies. However, radiation-related complications are still unavoidable risks for off-target cells. Little is known about radiation therapy's possible effects on mechanical features of the off-target cells such as human red blood cells (RBCs). RBCs are nucleus-free circulating cells that can deform without losing functionality in healthy conditions. Thus, to evaluate *in vitro* effects of radiation therapy on the healthy plasma membrane of cells, RBCs were selected as a primary test model. RBCs were exposed to clinically prescribed radiotherapy doses of 2 Gy, 12 Gy and, 25 Gy, and each radiotherapy dose group was compared to a non-irradiated group. Cells were characterized by stretching using dual-beam optical tweezers and compared using the resulting deformability index. The group receiving the highest radiation dose was found statistically distinguishable from the control group (DI_0Gy_ = 0.33 ± 0.08), and revealed the highest deformability index (DI_25Gy_ = 0.38 ± 0.11, *p* = 0.0068), while no significant differences were found for 2 Gy (DI_2Gy_ = 0.33 ± 0.08, *p* = 0.9) and 12 Gy (DI_12Gy_ = 0.31 ± 0.09, *p* = 0.2) dose groups. Based on these findings, we conclude that radiotherapy exposure may alter the deformability of red blood cells depending on the dose amount, and measurement of deformability index by dual-beam optical tweezers can serve as a sensitive biomarker to probe responses of cells to the radiotherapy.

## Abstract 2

Little is known about radiation therapy's possible effects on mechanical features of off-target cells such as human red blood cells. Here, irradiated human red blood cells were stretched using dual-beam optical tweezers and compared using the resulting deformability index.

## Graphical abstract

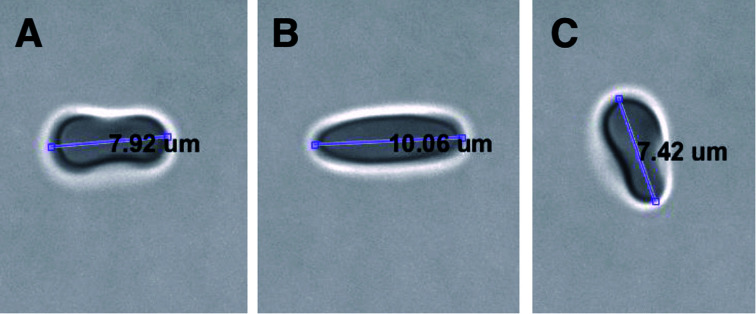

## Introduction

Radiation therapy is widely used as part of treatment for malignancies since it is capable of killing cancer cells and shrinking tumors using ionizing radiation.^1,2^ However, treatment with ionizing radiation typically faces issues not only because of the action on the target tumor but also because of the adverse influences of radiation exposure on non-target cells or tissues.^2–5^ In recent years, there has been an increasing amount of research on the use of ionizing radiation due to the high targeting capacity and therapeutic effect. Though radiation therapy brings many advantages, radiation-related complications are still unavoidable risks for off-target cells. Accordingly, decreasing its adverse consequences on off-target cells is a critical problem and remains a significant research area for radiation biology.^2–5^ Radiotherapy can promote biological damage at the molecular level and cellular DNA chains as single- or double-strand breaks.^6^ On the one hand, DNA in the nucleus was marked as the initial and fundamental target of radiation exposures: directly by deposition of energy or indirectly by producing reactive oxygen/nitrogen species. Thus, the primary adverse effects are attributed to DNA deterioration in target cells that have not been appropriately brought back through metabolic repairment.^6,7^ On the other hand, radiotherapy also acts directly on the membrane, confirming that plasma membrane shows an alternate path to DNA in radiation-modulated cell reactions.^8^ Previous studies have documented the direct influences of cellular death caused by human tissue irradiation, particularly chromosome rearrangement and genetic mutation resulting from the radiation-induced deposition of energy.^9–12^ Also, the effects of irradiation of RBCs on free hemoglobin level, sodium ions (Na^+^), potassium ions (K^+^), and chloride ions (Cl^−^) concentrations, a reduction in the production, and alterations in the aggregation state of platelets have been studied.^13–22^ These investigations mostly reveal an association between the irradiation dose and the loss of the red blood cell function.

However, little is known about the possible effects on mechanical features of red blood cells exposed to radiation. Measurement of mechanical properties such as elasticity, deformability, and stiffness in biological specimens is extensively regarded to reflect biological functions' variations. It can demonstrate the mechanical principles of organism units in numerous physiological or pathological states.^23,24^ Both morphology and cytoskeleton meshwork regulate red blood cell deformability and impairment. Abnormalities in the biconcave disc shape, the membrane, and the cytoskeleton indicate some red blood cell-related diseases.^25^ They give rise to characteristic illnesses such as diabetes mellitus,^26^ sickle cell anemia,^27–29^ malaria,^30^ and cancer.^31^ It is also well-known that radiotherapy leads to many complex and dynamic variations in cell outline along with cell membrane damage. For example, changes in the membrane permeability and the membrane-cytoskeleton structure may induce differences in the membrane, containing metabolic and behavioral changes, giving rise to cellular dysfunction.^32–34^ Therefore, studying cell mechanics at the whole-cell level contributes to the further understanding radiation-related damage.

Several methods have been used to assess cells' elasticity and deformability, including AFM, micro-needle manipulation, cell poking, magnetic tweezers, and microfluidic chips.^28,35–40^ However, they encounter fundamental obstacles to generalized applications. The tools have a high stiffness relative to the modulus of elasticity of the sample, and they are not capable of resolving minor differences in cell elasticity. Some of these methods can only reach a limited fragment of the cell due to the contact area. Accordingly, whole-cell elasticity cannot be directly measured.

Optical laser traps enable holding, manipulation, and characterization of various microscopic and nanoscopic materials.^41–44^ Optical forces exerted through light due to the transfer of optical momentum have been applied to investigate the elasticity of RBCs using two optical techniques: optical tweezers method and optical stretcher method.^45–49^ The dual-beam optical tweezers in stretching mode produces forces that bridge those produced through conventional optical tweezers. Up until now, the ability of dual-beam optical tweezers in stretching mode to produce a measurable deformation of the RBCs exposed to radiotherapy has never been tested. For this reason, we probed whether definitive or subtle changes in the deformability of the RBCs in response to radiotherapy can serve as a sensitive biomarker by dual-beam optical tweezers measurements. For this reason, human red blood cells were irradiated with radiation doses at several levels, and the effects of radiation therapy were investigated through optical tweezers by the deformability index analysis and a hemogram test for each irradiation dose in the study.

## Results

### Radiotherapy effects on deformability index

**Fig. 1:**
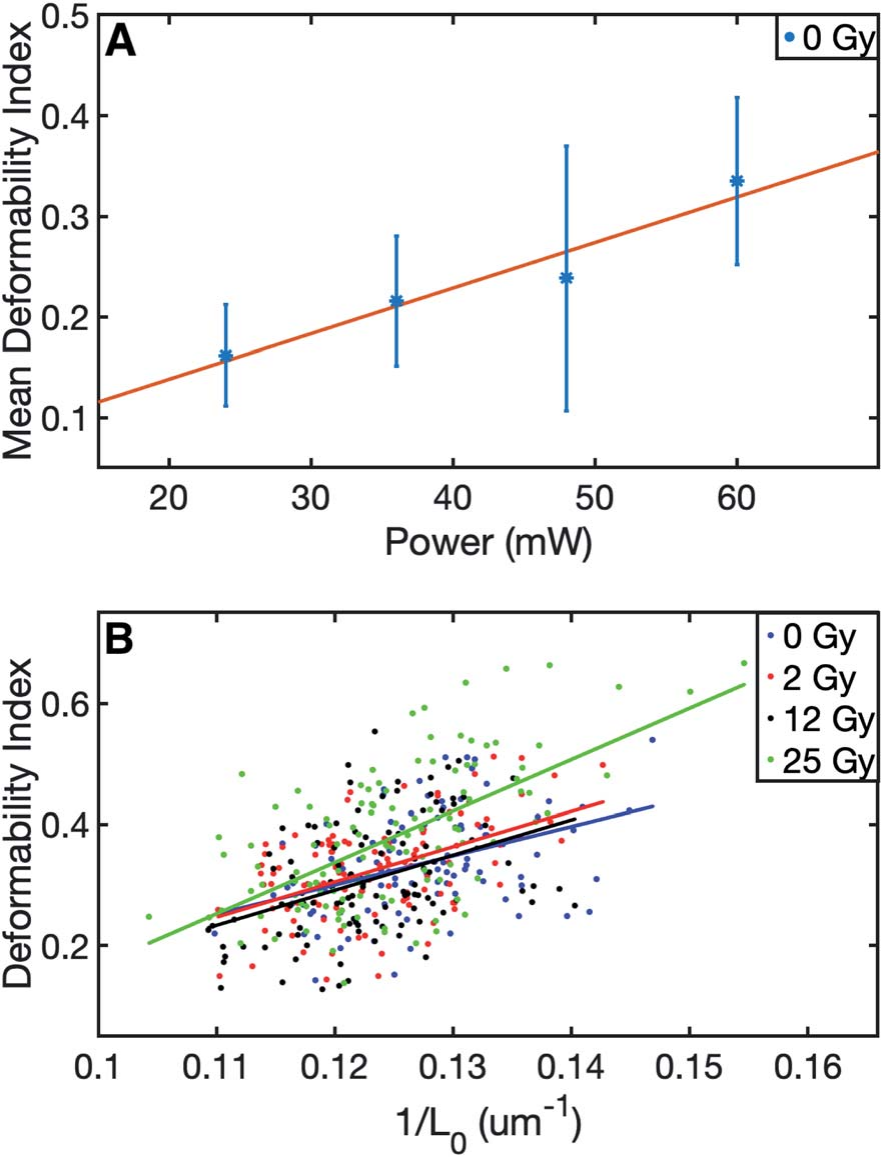
(A) Change in the mean DI of the control group for the laser trap powers operating at 24 mW, 36 mW, 48 mW, and 60 mW. The linear fit equation is *Y* = 0.0027*X* + 0.0475 with the *R*-squared value of 0.904. The error bars indicate standard errors, (B) the scatter plot of deformability index *vs.* inverse unstretched RBC size with the corresponding linear fit lines for the four groups; *Y*_0Gy_ = 4.8*X*_0Gy_ − 0.2, *Y*_2Gy_ = 5.8*X*_2Gy_ − 0.4, *Y*_12Gy_ = 5.8*X*_12Gy_ − 0.4, and *Y*_25Gy_ = 8.5*X*_25Gy_ − 0.7 with the corresponding *R*-squared values 0.164, 0.243, 0.156, 0.365 respectively.

**Fig. 2:**
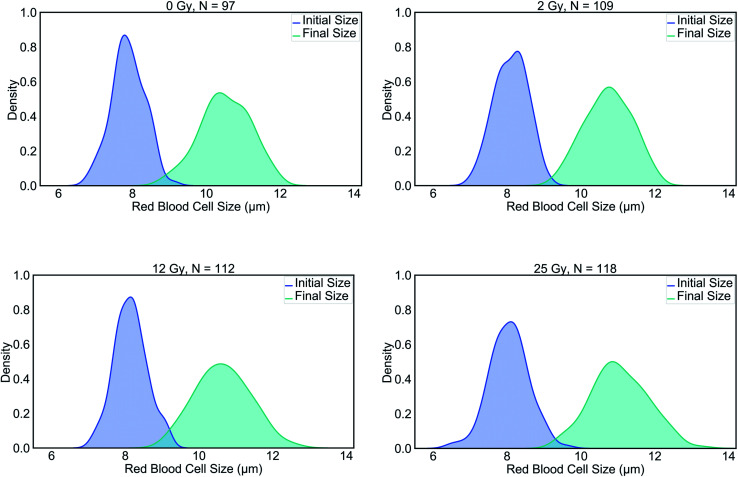
Kernel density estimations for pairs of initial and final RBC lengths in the control group, 2 Gy, 12 Gy and, 25 Gy groups.

**Fig. 3:**
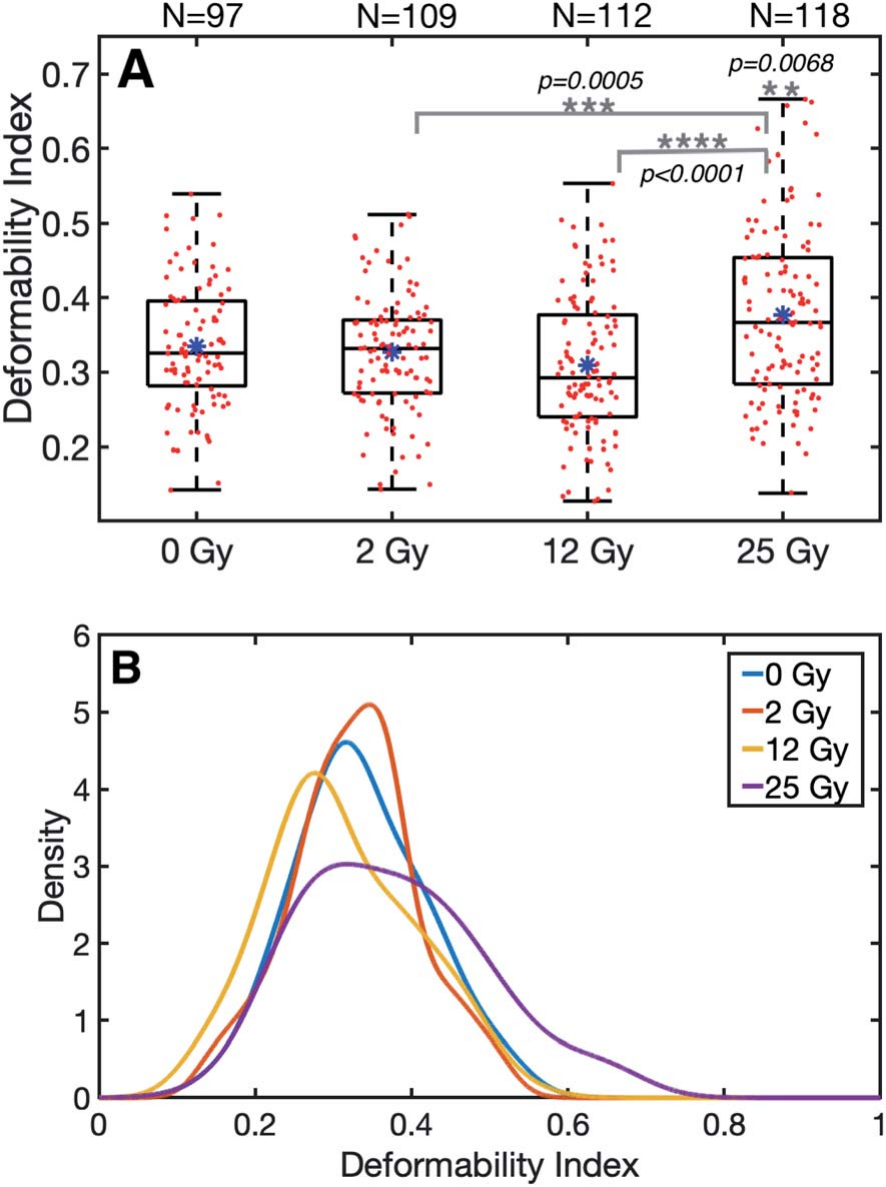
(A) The box plot shows the summary of the four data sets. The blue dot and the black line in the boxes show the mean and the median values of the corresponding data sets, respectively. Red dots represent the data points. (B) Kernel density estimations (kernel = normal) for DI are demonstrated for each group. Kernel bandwidths were found as: 0.0362, 0.0278, 0.0379, 0.0518 for the groups 0 Gy, 2 Gy, 12 Gy, 25 Gy, respectively.

The summary of the data can be viewed in Table 1. The results of this study evidenced significant differences in the final (stretched) cell size and deformability index of RBCs between the control and those with the highest level of radiation group. In terms of the initial (unstretched) cell size, all the dose groups significantly differed from the control group, whereas, in terms of the final cell size (*L*_max_), only the 25 Gy group exhibited a significant difference compared to all other groups (Table 2). The characterization of the dependence of DI on the laser power for the untreated cells revealed a linear relationship, as displayed in Fig. 1A.^50^ Since achieving the maximum stretching of the RBCs (without rupturing) was preferred, all the experiments were conducted with the laser traps operating at 60 mW total power at the trapping plane. The relation between the inverse unstretched size (1/*L*_0_) of RBCs and DI was analyzed by using linear regression. A linear relationship between the inverse unstretched cell size (1/*L*_0_) and DI was seen for all the groups (Fig. 1B). Especially the stretching amount (*L*_max_ − *L*_0_), which corresponds to the slope, was viewed to be elevated only for the 25 Gy dose group. This finding indicated that only the RBCs irradiated with 25 Gy dose were detected as more deformable than RBCs in the control group. Kernel density estimate plots, shown in Fig. 2, displayed normally distributed initial and final cell sizes for the four groups. The calculated deformability indexes for each data set were demonstrated in a box plot and the Kernel distribution plot, as shown in Fig. 3. Overall, the result of one-way ANOVA and Dunnett's multiple comparisons tests showed that relative to the control group, the mean DI of 25 Gy group was found to be increased, while no significant difference was detected for 2 Gy and 12 Gy dose groups. Among all the data sets, the highest variance in DI was observed in the 25 Gy group with the corresponding kernel bandwidth of 0.0518.

**Table 1:** The number of the measured RBCs, mean of *L*_0_, *L*_max_ and DI and the corresponding standard deviations are given for each data set

Dose (Gy)	# of RBCs	Mean *L*_0_ (μm)	Mean *L*_max_ (μm)	Mean DI
0	97	7.87 ± 0.44	10.51 ± 0.66	0.33 ± 0.08
2	109	8.11 ± 0.44	10.74 ± 0.44	0.33 ± 0.08
12	112	8.14 ± 0.43	10.65 ± 0.43	0.31 ± 0.09
25	118	8.05 ± 0.52	11.06 ± 0.76	0.38 ± 0.11

**Table 2:** Comparisons of the control group and experimental groups in accordance with the one-way ANOVA and Dunnett's multiple comparisons test. A *p* < 0.05 value was considered statistically different. ns shows no significant difference among groups

Dose groups (Gy)	*p*-Value		
	*L*_0_	*L*_max_	DI
0–2	0.004	0.09 (ns)	0.9 (ns)
0–12	0.0004	0.5 (ns)	0.2 (ns)
0–25	0.04	<0.0001	0.007
2–12	0.9 (ns)	0.8 (ns)	0.5 (ns)
2–25	0.8 (ns)	0.0040	0.0005
12–25	0.4 (ns)	<0.0001	2 × 10^−6^

### Radiotherapy effects on whole-blood count

Table 3 tabulates the hematological parameters in the non-irradiation (control) and the treated groups by 2 Gy, 12 Gy, and 25 Gy, respectively. It has been reported that in a complete blood count (CBC), the coefficient of variation between 3 to 20% (ref. 51) is expected. Therefore, CBC did not show radiation-related changes. Also, the blood components are not produced in the RBCs. Hence the variations in these components are not radiation-related but random.

**Table 3:** Whole blood count comparisons between the non-irradiated group and irradiated groups at 2 Gy, 12 Gy, and 25 Gy. WBC: white blood cell, RBC: red blood cell, HCT: hematocrit, MCH: mean corpuscular hemoglobin RDW: red cell distribution width, MCHC: mean cell hemoglobin concentration, MCV: mean cell volume, PLT: platelet, RDW-CV: Red Blood Cell Distribution Width. *L* in the parentheses means low with respect to the reference value

Parameters (unit)	Reference	Control	2 Gy	12 Gy	25 Gy
WBC (×10^3^ μL^−1^)	4.0–10.0	4.98	4.93	6.64	6.78
Neutrophil (×10^3^ μL^−1^)	2.00–7.00	1.89 (L)	1.98 (L)	3.68	3.71
Lymphocyte (×10^3^ μL^−1^)	0.80–4.00	2.61	2.50	2.36	2.42
Monocyte (×10^3^ μL^−1^)	0.12–1.20	0.32	0.31	0.48	0.49
Eosinophil (×10^3^ μL^−1^)	0.02–0.50	0.14	0.13	0.10	0.12
Basophil (×10^3^ μL^−1^)	0.00–0.10	0.02	0.01	0.02	0.04
RBC (×10^6^ μL^−1^)	3.50–5.50	4.50	5.06	4.53	4.50
HGB (g dL^−1^)	11.0–16.0	13.7	14.8	13.9	13.7
HCT (%)	37.0–54.0	43.8	45.7	44.0	43.8
MCV (fL)	80.0–100.0	97.2	90.3	97.1	97.2
MCH (pg)	27.0–34.0	29.6	29.2	30.6	30.5
MCHC (g dL^−1^)	32.0–36.0	32.9	32.3	31.5 (L)	31.4 (L)
RDW-CV (%)	11.0–16.0	12.7	11.8	12.8	12.7
RDW-SD (fL)	35.0–56.0	51.7	44.3	52.0	51.7
PLT (×10^3^ μL^−1^)	100–300	259	252	276	282

## Discussion

The deformability index analysis of human red blood cells, irradiated at 2 Gy, 12 Gy, and 25 Gy radiation doses, was indicated the significant distinction of small changes only in the deformability of RBCs irradiated by 25 Gy dose compared to the control group (*p* = 0.007). The possible reasons for the deformability change of RBCs due to the ionizing radiation are described next.

Radiobiology has been studying the biological responses of cells or tissues to ionizing radiation. It is well-known that ionizing radiation provokes damage directly by deposition of energy or indirectly by producing reactive oxygen/nitrogen species in DNA double helix in the nucleus. However, the origin of molecular and mechanical events triggered at the plasma membrane caused by radiation is unclear. In this study, RBCs were used as primary test objects since they are nucleus-free and have smooth membrane surfaces maintaining their integrity. In contrast, all white blood cells and platelets have nucleic acids that regulate cellular activities. Nucleic acids are reported to get adversely affected by radiation.^52^ Therefore, the presence of nucleic acids potentially has effects on cell deformability. However, we wanted to evaluate the effect of irradiation only on the cell membrane, and therefore, we focused our investigation on the red blood cells only. Since RBCs are nucleus-free and off-target cells circulating throughout the body, they are very suitable for studying the mechanical effects of radiation exposure on the membrane structure. Taken together, this study focused on any potential radiation-related effects on the deformability of the red blood cell cortex to distinguish these from adverse effects arising from DNA damage. Healthy red blood cells have good mechanical characteristics that build upon the biconcave disc shape, the cytoplasm viscosity, cytoskeleton and the stiffness of the phospholipid bilayer membrane. Current literature suggests that variation of any of these contributors may induce differences in the membrane morphology, cytoskeletal network, and mechanical features of RBCs and thus create morphologically distinct signals.^53,54^ As previously mentioned, these are responsible for both the deformability of red blood cells and variations in biological function. More specifically, notable variations in red blood cells arise from a coupled dynamic response of the membrane and spectrin meshwork. Spectrin, a fundamental component of cytoskeleton proteins, has not only a crucial role in preserving the cell outline and cytoskeleton network, but also controls deformability or responses to exterior perturbations.^55^ Our investigation suggests that radiation exposure of red blood cells by 25 Gy generated the highest deformability among the irradiated groups. Based on statistical analysis for deformability index, the red blood cell population for 25 Gy was significantly distinguishable with 95% confidence from the non-irradiated population. One of the reasons underlying this finding may be explained by the rearrangement of lipids, fatty acids, and spectrin-α1 protein and the collapse of the membrane skeleton due to the radiation effects on the plasma membrane.^56^ A link between cell stiffness and radiotherapy treatment was formerly documented. Therefore this difference was as anticipated.^57,58^ Besides, a study^59^ drew attention to the alterations observed in the intensities of actin and spectrin molecules. Those have essential roles in cell mechanics,^60–62^ as the determinant factor of cells' mechanical behavior in response to the radiation.

Moreover, the hemogram results showed that the number of RBCs, WBCs, and platelets did not decrease considerably and stayed in the reference range after being irradiated by 25 Gy and the remaining dose groups. These findings evidenced no radiation-related damage to the red blood cell's integrity during our experiments. As for WBCs in hemogram, the dose was not an influencing factor in the count after radiotherapy exposure. However, in the study of Sanzari *et al.*,^63^ radiotherapy application significantly decreased the number of white blood cells. Also, Taqi *et al.* reported no significant variations in the whole-blood count, and hence white blood cell counts.^63^ Any differences between the current investigations and the literature reports could be attributed to the differences in the deposited amount of the irradiation doses in the cells. Furthermore, hemoglobin concentration level was decreased in 25 Gy dose exposure compared to the non-irradiated group. This could be because the plasma membrane consists of unsaturated lipid and hemoglobin (free radical reaction sources). This may cause peroxidation of lipid, which can influence the fluidity and state of hemoglobin. Contrary to published studies,^57,58^ this study did not find a significant difference between 0 Gy and 2 Gy in terms of deformability of red blood cells. Overall, red blood cells responded to radiotherapy treatment differently because of the absorbed dose and cell features. While the definite mechanisms that alter the deformability index of RBCs due to ionizing radiation remain unclear, the results served in this study open a new window into the field of radiobiology. For further studies, together with deformability index analysis, Western blot analysis could be used to assess radiation-related cellular protein damage.

Furthermore, it is well known that irradiation inhibits lymphocyte proliferation and prevents transfusion-related graft-*versus*-host disease (TAGVHD).^64^ Although other leukocyte depletion techniques are often preferred,^65^ irradiation is still needed for transfusion of high risk-patient groups.^66^ The recommended irradiation dose of blood products is 25 Gy, and the recommended dose range is 15–50 Gy.^64^ While our results indicated that 25 Gy causes impairment of red blood cell deformability significantly, we emphasize that the replication of our findings needs further dedicated efforts. Therefore, the development of reliable lymphocyte depletion techniques that can be used instead of ionizing radiation is needed.

In terms of the homogeneity of the RBC data set, we also considered possible reticulocyte contributions. Immature red blood cells are called reticulocytes until they reach maturity. Unlike RBCs, they lack biconcave morphology, which affects their deformability adversely.^67^ Reticulocytes have a large nominal size and wider size distribution compared to mature RBCs^67,68^ They also contain remnant nucleic acids that stay functional and actively govern protein synthesis.^69^ Therefore, we expected ionizing radiation to induce chromosomal damage on the nucleic acids of reticulocytes in our samples. Radiation may result in micronuclei formation in reticulocytes, and in fact, reticulocyte micronucleus assays are widely used for assessing chromosomal damage.^70,71^ Micronucleus may act as a focal point of altered stiffness; therefore, it may have a physical effect in cell deformability. Additionally, altered protein synthesis may result in unpredictable alterations in cell deformability. Thus, aberrant deformability characteristics of reticulocytes with or without ionizing radiation damage can be considered a confounding factor in our deformability measurement method. However, we anticipated that only 0.5–2% of the RBC samples analyzed in this study would be reticulocytes^72^ since our controls do not have a disorder with effects on erythropoiesis. Furthermore, we had visually confirmed the biconcave morphology of the RBCs when we captured and rotated them during the optical stretching. Therefore, we considered only a negligible number of RBCs in our samples to be reticulocytes.

When the potential laser-induced heat damage risk on the RBCs during the trapping was regarded, we may say that this risk was minimized. Because the total laser power that the RBC was exposed to was low, and the experiment was conducted only once on an individual RBC. In this way, heat-related laser damage on RBCs was reduced.^73^ Secondly, laser absorption of cytoplasm, surrounding medium, and intracellular hemoglobin may cause an increase in the RBC temperature. Potentially this may affect the deformability of the RBC. However, 1064 nm Nd:YAG laser was utilized in this study, and at this wavelength, light absorption by water, intracellular proteins, and hemoglobin is shown to be minimal.^47,74^ Thirdly, since all the experiments were performed with the laser traps operating at 60 mW in 20 seconds, heat damage risk was further reduced. The analytical model shows that when using 100 mW laser tweezers at the wavelength of 1064 nm, the RBC temperature increases <13 K starting from 293 K and reaches to steady-state around 1 second.^74^ Therefore, we anticipate a temperature rise of <10 K from room temperature in our analysis. Lastly, temperature gradient rather than maximum temperature increase is another concern around laser absorption and heating in laser tweezers applications. Large temperature gradients may induce optoporation of the cell membrane,^75^ which would be undesired for the RBC deformability analysis. Experimental investigations showed that permeabilization damage of optically trapped RBCs due to temperature gradient was observed at trapping powers ≥280 mW at similar time scales, which is considerably higher than 60 mW used in our experiments. It is evident that laser tweezers may affect cellular processes through heat transfer. Nonetheless, we postulate that these effects are avoided in our deformability analysis by using the near-infrared laser wavelength at the safe power intensity and duration regime.

In conclusion, up until now, no study has measured the deformability of RBCs as a determinant factor to assess cell reactions to radiotherapy. We showed a feasibility study of radiation therapy effects on the mechanical properties of red blood cells in terms of deformability index using dual-beam optical tweezers in stretching mode. Dual-beam optical tweezers make it possible to perform cell stretching experiments directly without using microbeads. A significant benefit of the direct trapping technique over the method using microbead handles is that there is no requirement to attach and position the microbeads to the cortex of the RBCs for stretching measurements. With this, we were able to measure the whole-cell deformability of a large number of red blood cells in a life-span of cultured live-cells by eliminating the time taken to attach and locate the microbeads.

However, the main disadvantage of direct trapping and stretching without using the microbead handles is that the force calculation is complicated. In the case of stretching with the microbead handles, the force calibration is done on the microbeads. Hence the applied force on a cell due to the stretching can be calculated. However, in our case, the absence of the microbeads made the force calculation complicated. Since the stretching starts while the traps are in the cell, it stretches as the trap separation increases. It is unclear how and when the cell configures itself when the trap is moved in this situation. So that, for this study, we were unable to calculate the force on the cells.

Dual-beam optical tweezers also allowed us to probe definitive and subtle variations in the deformability of the red blood cells after radiotherapy treatment. Therefore, the findings of the present study provided the following insights for future research: deformability, as measured by dual-beam optical tweezers, may be used as a biomarker to follow changes in response to radiotherapy treatment in a cell's mechanics, that is shown up in the plasma membrane and cytoskeleton network in radiobiology investigations. Thus, this method may offer further knowledge in cellular function after radiotherapy with a more unifying view, containing mechanical properties from the membrane. Deformability measurements by the dual-beam optical tweezers may put a new emphasis on radiotherapy-induced effects in the future.

## Methods

### Collection and the transport of the blood samples

Two healthy women volunteered for this study with the ethical permission (2020/06) of Boǧaziçi University Science and Engineering Fields Human Research Ethics Committee (FMINAREK). The whole-blood from the participants was drawn by venipuncture and collected in 6 mL vacutainer Ethylene Diamine Tetra Acetic Acid (EDTA) tubes. Then, after separating the control group tubes, the remaining samples were sent to the radiology department for the irradiation procedure. The irradiation was performed within 2 h after the blood collection. Afterward, the irradiated blood samples were delivered to the laboratory within a transport bag kept at 4 °C. All the measurements were completed within two days after blood collection.

### Sample preparation

Whole blood of 0.1 μL was mixed with a solution consisting of 1 mL PBS (phosphate-buffered saline) and 100 μL BSA (bovine serum albumin) in an eppendorf tube. 70 μL of the sample was placed onto the microscope slide, and BSA-dried cover glass was placed on top. The edges of the cover glass were sealed using nail polish. The BSA-dried cover glass was prepared by placing 15 μL BSA on a cover glass and kept in an incubator at 24.5 °C for 2 hours.^76^ This process was done to prevent RBCs from adhering to the cover glass and make them floating in the solution. All the experiments were performed at room temperature.

### Irradiation protocol

The blood samples stored in EDTA tubes were irradiated by a total and a single-dose application of 2 Gy, 12 Gy, and 25 Gy in a single fraction by LINAC (Elekta Versa HD, Elekta, Crawley, UK) in Istanbul Oncology Hospital. This study used 6 MV flat with a dose rate of 600 cGy min^−1^ used in *d*_max_ and unflattened photon beams with 1200 cGy min^−1^ dose rate. The EDTA tubes were submerged in a custom-made rice phantom to distribute radiation homogeneously and mimic the body. The phantom was scanned through Computer Tomography (CT) with a slice thickness of 2 mm before treatment. The EDTA tubes with blood samples were defined as a Gross Tumor Volume (GTV). Monaco Treatment Planning System took over the dose distributions of the current study. The EDTA tubes filled with blood samples were considered as a GTV. Through recommended doses using fields of radiation Anterior–Posterior/Posterior–Anterior (AP/PA), the volume of the gross tumor volume complied with 95/100 (95%). Using collapsed cone algorithm Treatment Planning System (Monaco 5.11.02v, TPS), the motor unit was calculated. The monitor unit number was determined using 10 cm × 10 cm fields through the collapsed cone calculation technique to consider inhomogeneity to deliver the radiation doses of 2 Gy, 12 Gy, and 25 Gy to the red blood cell solutions kept in the tubes. LINAC output was corrected to confirm differences in doses at *d*_max_ smaller than 2% differences following TRS 398 (ref. 77) prior to the irradiation. Irradiation doses were verified for each experiment by 0.6 cc farmer-type ionization chamber. The rice phantom position was checked to confirm the tubes and the tomography plan were observed in the same region. For each irradiation, the position accuracy of the tubes inside the rice phantom was controlled by comparing DRR and kV images.^78^

### Experiment and analysis

**Fig. 4:**
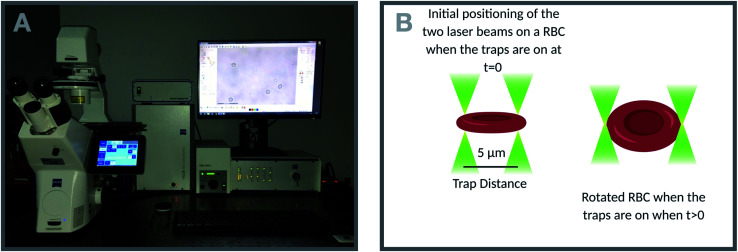
(A) The dual-beam optical tweezers set-up used to quantify deformability of RBCs after radiotherapy treatment, (B) a cartoon of initial positioning of the laser traps on an RBC in stretching mode.

**Fig. 5:**
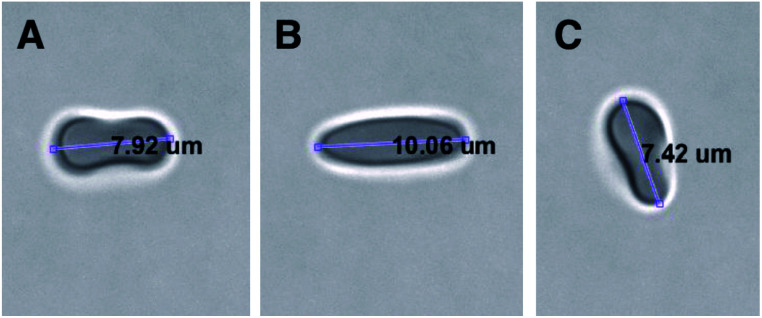
Top view of stretching of an RBC by the two optical traps; (A) the traps were positioned on the two ends of the RBC with the trap separation of 5 μm, (B) the RBC was reached to its maximum length just before escaping from the moving trap, (C) the relaxed RBC after escaping from the moving trap. A video of the experiment was provided as ESI (Video S1†).

A commercial dual-beam tweezers (Zeiss PALM Micro Tweezers) with 1064 nm wavelength Nd:YAG laser, 3 W output power, and 100× oil immersion (NA = 1.518) objective was used to perform the stretching experiments, see Fig. 4A. According to the power measurement with a power meter on the trapping plane, we found that the power of the traps operating at 100% corresponds to 60 mW. Since the total laser power of the optical traps could be tunable in terms of percentage power on the user interface, the stretching experiments were performed with different trap powers (operating at 40%, 60%, 80%, and 100%) on the control group (0 Gy) to characterize the dependence of DI on the trapping laser power (Fig. 1A). Then, all the experiments were conducted with the laser power operating at 100% (30 mW in each trap at the trap location). The stretching experiment was constructed by setting the determined parameters (velocity, movement direction and the power of the traps, and the experiment duration) on the user interface of the tweezers. With this setting, the experiment was automatically performed by the tweezers. Before starting the experiment, first, while the traps were off, they were positioned on the two ends of the RBC with the trap separation of 5 μm. When the traps were activated, the experiment was started. During the stretching, one of the traps was moving with the velocity of 0.5 μm s^−1^ for 20 s automatically by the tweezers, while the other was kept fixed in position. With the movement of the trap, the RBC first started stretching, and then after reaching the maximum stretched length, it escaped from the moving trap and began to relax (Fig. 5). For each dose, the experiment was performed on the day of radiation treatment and the one day after to check if there is a time-dependent radiation effect on the deformability of the RBCs. The experiment was performed once on an individual RBC.

Deformability index analysis in this study relies on computer vision techniques. Therefore the spatial resolution of our method is limited by the size of a single-pixel which corresponds to 70 nm. It should also be noted here that the frame rate of the recordings is 10 fps, and we moved the optical traps at the speed of 0.5 μm s^−1^. As a result, it can be concluded that the resolution is not limited by the recording rate. When the one-pixel size is considered as the minimum difference between the initial and final length of an RBC, the spatial resolution of 70 nm corresponds to the minimum detectable DI of 0.009 for an RBC of the average size in our experiments. In the analysis, a MATLAB code was used to calculate the axial diameter of the cell during the stretching. The algorithm of the code for the analysis of one cell can be summarized in four steps: (1) all the frames of the cell recorded in 20 s are converted into grayscale, (2) using an automatic grayscale threshold, the grayscale images were turned into binary images, (3) maximum Feret diameter (MFD) function was used to find the edge and the axial length of the cell in each frame, (4) maximum (*L*_max_) and the minimum (*L*_0_) values of the MFD were extracted, and DI was calculated using the following equation:^49^1
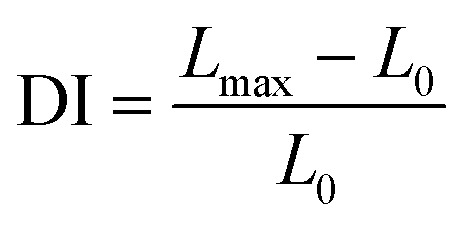
where, *L*_0_ is the unstretched (initial) size, *L*_max_ is the maximum (final) stretched size of the RBC. For all doses, no significant differences were found in DI according to the two measurement days (radiation treatment day and the day after). In addition to this, no significant difference was observed between the DI of the two persons. Therefore the all the data sets were grouped in the analysis in terms of the radiation dose only. By considering DI as the dependent variable and *L*_0_ as the independent variable, univariate linear regression analysis was achieved (Fig. 1B). Statistical differences were calculated using one-way ANOVA and Dunnett's multiple comparisons test.

## Author contributions

M. B. U. initiated and constructed this study. M. T. I. and I. D. performed the dual-beam optical tweezers measurements, M. T. I. and I. D. prepared the red blood cell samples. C. C. and A. O. performed the radiotherapy treatment and hemogram analysis. U. A. G., U. G., C. C., A. O., and O. G. analyzed the corresponding findings. M. T. I., I. D., and U. G. wrote the manuscript. All authors reviewed the paper.

## Conflicts of interest

There are no conflicts to declare.

## Supplementary Material

RA-011-D1RA01948A-s001

## References

[cit1] Chen H. H., Kuo M. T. (2017). Oncotarget.

[cit2] De Ruysscher D., Niedermann G., Burnet N. G., Siva S., Lee A. W., Hegi-Johnson F. (2019). Nat. Rev. Dis. Primers.

[cit3] Umegaki K., Sugisawa A., Shin S. J., Yamada K., Sano M. (2001). Free Radicals Biol. Med..

[cit4] Marín A., Martín M., Liñán O., Alvarenga F., López M., Fernández L., Büchser D., Cerezo L. (2015). Rep. Practical Oncol. Radiother..

[cit5] Wang J.-s., Wang H.-j., Qian H.-l. (2018). Mil. Med. Res..

[cit6] Georgakilas A. G. (2008). Mol. BioSyst..

[cit7] Georgakilas A. G., O'Neill P., Stewart R. D. (2013). Radiat. Res..

[cit8] Corre I., Niaudet C., Paris F. (2010). Mutat. Res., Rev. Mutat. Res..

[cit9] Wang Y., Liu L., Pazhanisamy S. K., Li H., Meng A., Zhou D. (2010). Free Radicals Biol. Med..

[cit10] Nomura T., Hongyo T., Nakajima H., Li L. Y., Syaifudin M., Adachi S., Ryo H., Baskar R., Fukuda K., Oka Y. (2008). et al.. Mutat. Res., Genet. Toxicol. Environ. Mutagen..

[cit11] Gavara N., Chadwick R. S. (2012). Nat. Nanotechnol..

[cit12] Coates P. J., Lorimore S. A., Wright E. G. (2004). Mutat. Res., Fundam. Mol. Mech. Mutagen..

[cit13] Liumbruno G., Bennardello F., Lattanzio A., Piccoli P., Rossetti G. (2009). Blood Transfus..

[cit14] Brugnara C., Churchill W. (1992). Transfusion.

[cit15] Dinning G., Doughty R., Reid M., Lloyd H. (1991). Br. Med. J..

[cit16] Chapman J., Finney R., Forman K., Kelsey P., Knowles S., Napier J., Phillips P., Mitchell R., Murphy M., Waters A. (1996). et al.. Transfusion Medicine.

[cit17] Rivet C., Baxter A., Rock G. (1989). Transfusion.

[cit18] Agarwal P., Ray V., Choudhury N., Chaudhary R. (2005). Indian J. Med. Res..

[cit19] Jin M., Jeon H., Jung H. J., Kim B., Shin S. S., Choi J. J., Lee J. K., Kang C.-Y., Kim S. (2003). Exp. Biol. Med..

[cit20] Li W., Wang G., Cui J., Xue L., Cai L. (2004). Exp. Hematol..

[cit21] Peslak S. A., Wenger J., Bemis J. C., Kingsley P. D., Frame J. M., Koniski A. D., Chen Y., Williams J. P., McGrath K. E., Dertinger S. D. (2011). et al.. Exp. Hematol..

[cit22] Moroni M., Elliott T. B., Deutz N. E., Olsen C. H., Owens R., Christensen C., Lombardini E. D., Whitnall M. H. (2014). Int. J. Radiat. Biol..

[cit23] Xu W., Mezencev R., Kim B., Wang L., McDonald J., Sulchek T. (2012). PLoS One.

[cit24] Tomaiuolo G. (2014). Biomicrofluidics.

[cit25] Weng H., Guo X., Papoin J., Wang J., Coppel R., Mohandas N., An X. (2014). Biochim. Biophys. Acta, Biomembr..

[cit26] Buys A. V., Van Rooy M.-J., Soma P., Van Papendorp D., Lipinski B., Pretorius E. (2013). Cardiovasc. Diabetol..

[cit27] Maciaszek J. L., Lykotrafitis G. (2011). J. Biomech..

[cit28] Man Y., Kucukal E., An R., Watson Q. D., Bosch J., Zimmerman P. A., Little J. A., Gurkan U. A. (2020). Lab Chip.

[cit29] Alapan Y., Little J. A., Gurkan U. A. (2015). Sci. Rep..

[cit30] An X., Mohandas N. (2010). Transfus. Clin. Biol..

[cit31] Katira P., Zaman M. H., Bonnecaze R. T. (2012). Phys. Rev. Lett..

[cit32] Park H. J., Griffin R. J., Hui S., Levitt S. H., Song C. W. (2012). Radiat. Res..

[cit33] Garaj-Vrhovac V., Gajski G., Pažanin S., Šarolić A., Domijan A.-M., Flajs D., Peraica M. (2011). Int. J. Hyg. Environ. Health.

[cit34] Lara P. C., López-Peñalver J. J., de Araújo Farias V., Ruiz-Ruiz M. C., Oliver F. J., de Almodóvar J. M. R. (2015). Cancer Lett..

[cit35] Felder S., Elson E. L. (1990). J. Cell Biol..

[cit36] Daily B., Elson E. L., Zahalak G. I. (1984). Biophys. J..

[cit37] Radmacher M. , Fritz M., Kacher C. M., Cleveland J. P. and Hansma P. K., Measuring the viscoelastic properties of human platelets with the atomic force microscope, 1996 10.1016/S0006-3495(96)79602-9 PMC1224955 8770233

[cit38] Dao M., Lim C. T., Suresh S. (2003). J. Mech. Phys. Solids.

[cit39] Chen J., Fabry B., Schiffrin E. L., Wang N. (2001). Am. J. Physiol.: Cell Physiol..

[cit40] MacKay J. L. and Kumar S., Cell Imaging Techniques, Springer, 2012, pp. 313–329

[cit41] Ashkin A. (1970). Phys. Rev. Lett..

[cit42] Ashkin A., Dziedzic J. M., Yamane T. (1987). Nature.

[cit43] Maragò O. M., Jones P. H., Gucciardi P. G., Volpe G., Ferrari A. C. (2013). Nat. Nanotechnol..

[cit44] Polimeno P., Magazzu A., Iatì M. A., Patti F., Saija R., Boschi C. D. E., Donato M. G., Gucciardi P. G., Jones P. H., Volpe G. (2018). et al.. J. Quant. Spectrosc. Radiat. Transfer.

[cit45] Liao G.-B., Bareil P. B., Sheng Y., Chiou A. (2008). Opt. Express.

[cit46] Guck J., Ananthakrishnan R., Mahmood H., Moon T. J., Cunningham C. C., Käs J. (2001). Biophys. J..

[cit47] Henon S., Lenormand G., Richert A., Gallet F. (1999). Biophys. J..

[cit48] Rancourt-Grenier S., Wei M.-T., Bai J.-J., Chiou A., Bareil P. P., Duval P.-L., Sheng Y. (2010). Opt. Express.

[cit49] Agrawal R., Smart T., Nobre-Cardoso J., Richards C., Bhatnagar R., Tufail A., Shima D., Jones P. H., Pavesio C. (2016). Sci. Rep..

[cit50] De Luca A. C., Rusciano G., Ciancia R., Martinelli V., Pesce G., Rotoli B., Selvaggi L., Sasso A. (2008). Opt. Express.

[cit51] Vis J., Huisman A. (2016). International Journal of Laboratory Hematology.

[cit52] Teoule R. (1987). Int. J. Radiat. Biol. Relat. Stud. Phys., Chem. Med..

[cit53] Heinrich V., Ritchie K., Mohandas N., Evans E. (2001). Biophys. J..

[cit54] Švelc T., Svetina S. (2012). Cell. Mol. Biol. Lett..

[cit55] Haghparast S. M. A., Kihara T., Shimizu Y., Yuba S., Miyake J. (2013). J. Biosci. Bioeng..

[cit56] Haimovitz-Friedman A., Kan C.-C., Ehleiter D., Persaud R. S., Mcloughlin M., Fuks Z., Kolesnick R. N. (1994). J. Exp. Med..

[cit57] Zhang B., Liu B., Zhang H., Wang J. (2014). PLoS One.

[cit58] Spyratou E., Dilvoi M., Patatoukas G., Platoni K., Makropoulou M., Efstathopoulos E. P. (2019). Journal of medical physics.

[cit59] Benderitter M., Vincent-Genod L., Pouget J., Voisin P. (2003). Radiat. Res..

[cit60] Nans A., Mohandas N., Stokes D. L. (2011). Biophys. J..

[cit61] Trepat X., Deng L., An S. S., Navajas D., Tschumperlin D. J., Gerthoffer W. T., Butler J. P., Fredberg J. J. (2007). Nature.

[cit62] Byers T. J., Branton D. (1985). Proc. Natl. Acad. Sci. U. S. A..

[cit63] Sanzari J. K., Wan X. S., Krigsfeld G. S., Wroe A. J., Gridley D. S., Kennedy A. R. (2013). Gravitational and space research, publication
of the American Society for Gravitational and Space Research.

[cit64] Rosen N., Weidner J., Boldt H., Rosen D. (1993). Transfusion.

[cit65] Dunbar L. N., Coleman Brown L., Rivera D. R., Hartzema A. G., Lottenberg R. (2012). Int. Scholarly Res. Not..

[cit66] Pritchard A. E., Shaz B. H. (2016). Arch. Pathol. Lab. Med..

[cit67] Malleret B., Xu F., Mohandas N., Suwanarusk R., Chu C., Leite J. A., Low K., Turner C., Sriprawat K., Zhang R. (2013). et al.. PLoS One.

[cit68] Brugnara C. (1998). Int. J. Clin. Lab. Res..

[cit69] Gierer A. (1963). J. Mol. Biol..

[cit70] MacGregor J. T., Bishop M. E., McNamee J. P., Hayashi M., Asano N., Wakata A., Nakajima M., Saito J., Aidoo A., Moore M. M. (2006). et al.. Toxicol. Sci..

[cit71] Abramsson-Zetterberg L., Grawé J., Zetterberg G. (1999). Mutat. Res., Fundam. Mol. Mech. Mutagen..

[cit72] Van den Bossche J., Devreese K., Malfait R., Van de Vyvere M., Wauters A., Neels H., De Schouwer P. (2002). Clin. Chem. Lab. Med..

[cit73] Zhong M.-C., Wei X.-B., Zhou J.-H., Wang Z.-Q., Li Y.-M. (2013). Nat. Commun..

[cit74] Krasnikov I., Seteikin A., Bernhardt I. (2011). J. Biophotonics.

[cit75] Chowdhury A., Waghmare D., Dasgupta R., Majumder S. K. (2018). J. Biophotonics.

[cit76] Zhang Z. W., Neu B. (2009). Biophys. J..

[cit77] Andreo P., Huq M. S., Westermark M., Song H., Tilikidis A., DeWerd L., Shortt K. (2002). Phys. Med. Biol..

[cit78] Demirkan I., Yaprak G., Ceylan C., Algul E., Tomruk C. O., Bilen B., Unlu M. B. (2020). Radiat. Oncol..

